# Health literacy interventions for pregnant women with limited language proficiency in the country they live in: a systematic review

**DOI:** 10.1186/s12889-024-20747-8

**Published:** 2024-11-26

**Authors:** Marya Khan, Arti Dave, Madeleine Benton, Ngawai Moss, Mandeep Kaur Kaler

**Affiliations:** 1 The Royal London Hospital, Whitechapel Road, London, E1 1FR UK; 2 Department of Psychological Medicine, Kings College London, London, UK; 3 Elly Charity, London, UK

**Keywords:** Health literacy, Pregnancy, Limited language proficiency, Migrant, Interventions, Systematic review

## Abstract

**Background:**

Low health literacy can present significant risks throughout pregnancy, impacting both maternal and fetal health outcomes. Those who do not speak the main language of their country of residence are more likely to have lower health literacy. Considering the heightened challenges faced by this demographic in accessing, understanding, and engaging with health information and services, designing and implementing tailored interventions is crucial to mitigate health disparities. This review aims to identify and examine health literacy interventions developed for pregnant women whose first language differs from the language predominantly spoken in their residing country.

**Methods:**

Electronic databases of Embase and Medline were searched using relevant search terms from their inception to July 2023. Data were extracted and analysed using narrative synthesis.

**Results:**

Of the 1964 identified records, three were included. The studies were conducted in Australia and Denmark, and ranged in design, including: a cluster randomised controlled trial, mixed methods design; and qualitative design. Health literacy intervention modalities included midwifery education combined with a smartphone application and leaflet, culturally adapted group classes, and informative videos. The interventions were translated into various languages to cater to the target populations. Two studies used the Health Literacy Questionnaire to assess the intervention effectiveness, yielding contrasting results: one showed no improvement, while the other reported increased post-intervention health literacy scores. The third had not yet evaluated intervention effectiveness.

**Conclusions:**

This review identifies a significant scarcity in health literacy interventions for pregnant women whose first language differs to the predominant language of the country they live in, despite their greater need for support. While few studies were found, their diversity suggests multiple strategies for enhancing health literacy. Bridging this health literacy gap for linguistically diverse pregnant populations could reduce disparities in maternal and fetal outcomes, underscoring the need for targeted, evaluated interventions that actively engage affected women and their support networks.

**Trial registration:**

Registered with PROSPERO: CRD42023475511. Date of registration: 10.11.23.

**Supplementary Information:**

The online version contains supplementary material available at 10.1186/s12889-024-20747-8.

## Background

Health literacy is integral in enabling individuals to actively manage and make informed decisions about their health, as well as navigate often complex healthcare systems [1]. The health literacy literature has rapidly expanded, yet there remains a lack of consensus as to a single gold-standard definition [2, 3]. A recent systematic review defined health literacy as ‘the ability of an individual to obtain and translate knowledge and information in order to maintain and improve health in a way that is appropriate to the individual and system contexts’ [4]. Three key elements which encompass the concept of health literacy have been identified: (1) knowledge of health, healthcare, and health systems; (2) processing and using information in various formats in relation to health and healthcare; and (3) ability to maintain health through self-management and working in partnerships with health providers [4]. An evolved definition of health literacy which highlights a distinction between ‘personal health literacy’ and ‘organisational health literacy’ has also been proposed. This definition emphasises that the responsibility for health literacy does not rely solely on the individual, and that organisations must be equitable in how they address health literacy [5].

It has been consistently recognised that low levels of health literacy are associated with adverse health outcomes such as reduced uptake of preventive care, increased emergency department visits, increased hospitalisations, and increased mortality rates [6]. Health literacy, closely tied to various socioeconomic determinants of health [7], is proposed to play a partial mediating role in the connection between these determinants and observed health outcomes, contributing to health inequities [8].

Health literacy is especially crucial during pregnancy, as a pregnant woman’s health behaviours and decisions can profoundly impact outcomes for both them and the developing fetus [9]. There is information from a variety of sources [10] (i.e. antenatal classes) and a plethora of opportunities where women can be provided with recommendations regarding the initiation, maintenance and cessation of health behaviours [11], to optimise outcomes and prevent complications in pregnancy. Nevertheless, differences in maternal health literacy can impact one’s abilities to access and utilise this wealth of information effectively. Pregnancy has been considered a pivotal ‘teachable moment’ [12] for implementing interventions to enhance health behaviour, owing to universally high levels of patient activation and motivation [13].

A systematic review conducted by Nawabi et al. reported on associations between health literacy and outcomes in pregnancy [14]. Limited health literacy in pregnancy was associated with higher risk perception and negative beliefs regarding medication, as well as non-adherence to prescribed medication [15], including poor uptake of antidepressants and flu vaccinations [16]. In contrast, adequate health literacy was associated with increased likelihood of making an informed choice concerning prenatal genetic testing, which, in turn, correlated with reduced levels of decisional conflict and anxiety [17]; higher levels of health literacy were associated with more health promoting lifestyles, a planned pregnancy [16] and increased knowledge of age-related pregnancy risks [18].

In other smaller scale studies, higher health literacy levels have been associated with maintaining exclusive breastfeeding and serve as a protective factor against early breastfeeding cessation [19–21]. They are also associated with greater knowledge and utilisation of maternal healthcare services [22–25], increased knowledge and early detection behaviours of pre-eclampsia [26, 27], and increased knowledge of appropriate drug utilisation [28]. In contrast, low health literacy levels in pregnancy are associated with continued smoking [29] and tobacco consumption [30], self-efficacy barriers to information seeking [31], reduced likelihood of taking folic acid during pregnancy [32], and poor glycaemic control in populations with gestational diabetes mellitus [33]. In the secondary analysis of a larger multicentre cohort study, inadequate health literacy was associated with adverse maternal and infant outcomes, including a greater risk of caesarean birth, major perineal laceration, small-for-gestational-age status, low birth weight and low 5-minute Apgar score [34]. Hence, implementing interventions aimed at improving health literacy could reduce health disparities in maternal and neonatal outcomes.

A recent systematic review examining the effectiveness of health literacy interventions in general pregnant populations found limited interventions, with evidence suggesting an increase in health-related knowledge when implemented [35]. However, drawing meaningful conclusions on the impact of these interventions on other pregnancy outcomes was challenging due to small sample sizes and varied outcomes [35].

The literature has demonstrated particularly inadequate health literacy levels in both general and pregnant immigrant / migrant populations [36–40]. Additionally, a link between limited language proficiency and low health literacy has also been reported in the literature [41–43]. A study in 2018 demonstrated that 51% of migrants in the UK reported a language other than English as their first language at home [44]; moreover, limited language proficiency is reported to be one of the key determinants of the widely documented low levels of health literacy found in immigrant / migrant populations [45, 46]. In the context of pregnancy, immigrant women are often observed to experience higher rates of certain obstetric complications compared to their native counterparts. A systematic review identified higher incidence of stillbirth, early neonatal death, maternal death, as well as increased incidence of postpartum depression in some immigrant groups [47]. Another systematic review and meta-analysis found risk of emergency caesarean section, shoulder dystocia, gestational diabetes mellitus, small-for-gestational-age, 5-min Apgar less than 7 and oligohydramnios in the immigrant women to be significantly higher than in those with a native-origin background [48]. Studies in the United States have demonstrated that pregnant women with limited English proficiency are at increased risk of adverse outcomes in all stages of pregnancy [49], including higher rates of obstetric trauma during vaginal birth and potentially high-risk births [50]. The lower levels of health literacy found in these populations likely directly contributes to the increased risk of some adverse perinatal outcomes.

There is a clear need to improve healthcare equity for culturally and linguistically diverse pregnant people, with potential improvement through interventions targeting health literacy [51]. The primary aim of this review is to identify and examine health literacy interventions for pregnancy, developed specifically for women whose first language differs from the language predominantly spoken in their country of residence, covering the prenatal, perinatal and postnatal periods up to one year after birth. The secondary aims are to describe methods used to evaluate intervention effectiveness, and the outcomes of these interventions for women.

## Methods

### Design

The current systematic review was conducted in accordance with the Preferred Reporting Items for Systematic Reviews and Meta-Analyses (PRISMA) guidelines [52]. The review was conducted with health care professionals, researchers, and members of a patient and public involvement group. One public advisor contributed to the write-up of this work (NM). The protocol was registered on PROSPERO prospectively (ID: CRD42023475511).

### Search strategy

To identify potentially relevant articles, a systematic search of the databases Embase and Medline was conducted using Ovid. Search terms were developed in collaboration with the research team, research librarian, and based on previous literature and similar systematic reviews [14, 35]. Key words of health literacy, pregnancy, obstetric, prenatal and postnatal were used to capture our results. The full search strategy was “health literacy” AND “pregnan*” OR “maternal” OR “obstetric*” OR “postnatal*” OR “prenatal*”. The search was carried out from inception of the databases to July 2023. Two independent reviewers (MKh and AD) also engaged in a manual search on Google Scholar, as well as handsearching of references of the articles to be included, in attempts to identify any further relevant studies.

### Eligibility criteria

The eligibility criteria were developed using the Population, Intervention, Comparison, Outcome, Study design (PICOS) [53] framework. The inclusion criteria were health literacy interventions for pregnant women whose first language was not the predominant language spoken in the country where the study was being conducted. For the purposes of this review, health literacy was defined as ‘the ability of an individual to obtain and translate knowledge and information in order to maintain and improve health in a way that is appropriate to the individual and system contexts’ [4]. An intervention was any activity undertaken with the objective of improving human health [54]. Interventions that targeted pregnancy up to 1 year postnatal were included. Exclusion criteria were papers not published in English, and not from peer reviewed journals. We excluded any health literacy interventions that were only developed in the language predominantly spoken in the country where the study took place, which was not the first language of the target audience. For studies with resources in both the dominant and non-dominant languages, we included them only if data for the non-dominant language group were reported separately.

### Study selection and data extraction

Titles and abstracts of the identified articles were imported into Rayyan software to enable independent review. Two reviewers (MKh and AD) independently screened all 1364 abstracts. Full text articles of studies retained after the abstract screening process were retrieved and screened by the same two independent reviewers (MKh and AD). Disagreements were discussed and resolved via consensus with a third reviewer (MKa). Data were extracted from included articles using a structured and piloted data extraction form, by MKh and AD independently and cross checked by MB.

### Quality assessment

A formal quality appraisal of each included paper was conducted. The quality of included articles was assessed using the Mixed Methods Appraisal Tool (MMAT) [55]. The MMAT can be applied to five categories of studies: qualitative research, randomised controlled trials, quantitative non-randomised studies, quantitative descriptive studies and mixed method studies. This tool was deemed appropriate for quality appraisal of the studies included in our review, as they consisted of a combination of both qualitative and quantitative studies. The MMAT provides criteria for high quality evidence for each category of study. It should be noted, however, that in contrast to previous versions of the MMAT, the MMAT version 2018 has no clear guidance on how each study should be stratified based on the number of criteria met. The user guide advises against calculating an overall score [55]. Thus, an arbitrary, subjective overall rating was applied to each study based on the criteria met. Each paper included in the review was graded as either low, moderate or high quality by two reviewers (MKh and AD) independently. A third reviewer (MKa) was available if there were any discrepancies in the decisions. A more detailed presentation of how each MMAT criterion was rated has been summarised in a table from the MMAT version 2018 user guide [55] and can be found as supplementary material – see Supplementary Table 1, Additional file 1.

### Synthesis

A narrative synthesis [56] was undertaken by two authors (MKh and AD). Data was analysed by extracting key content from all papers regarding the intervention. Key elements extracted were the design of the intervention, any theories underlying the design, the target population, the delivery of the intervention, and any outcome measures. Comparisons were made between different interventions to identify common findings and key differences.

## Results

**Fig. 1:**
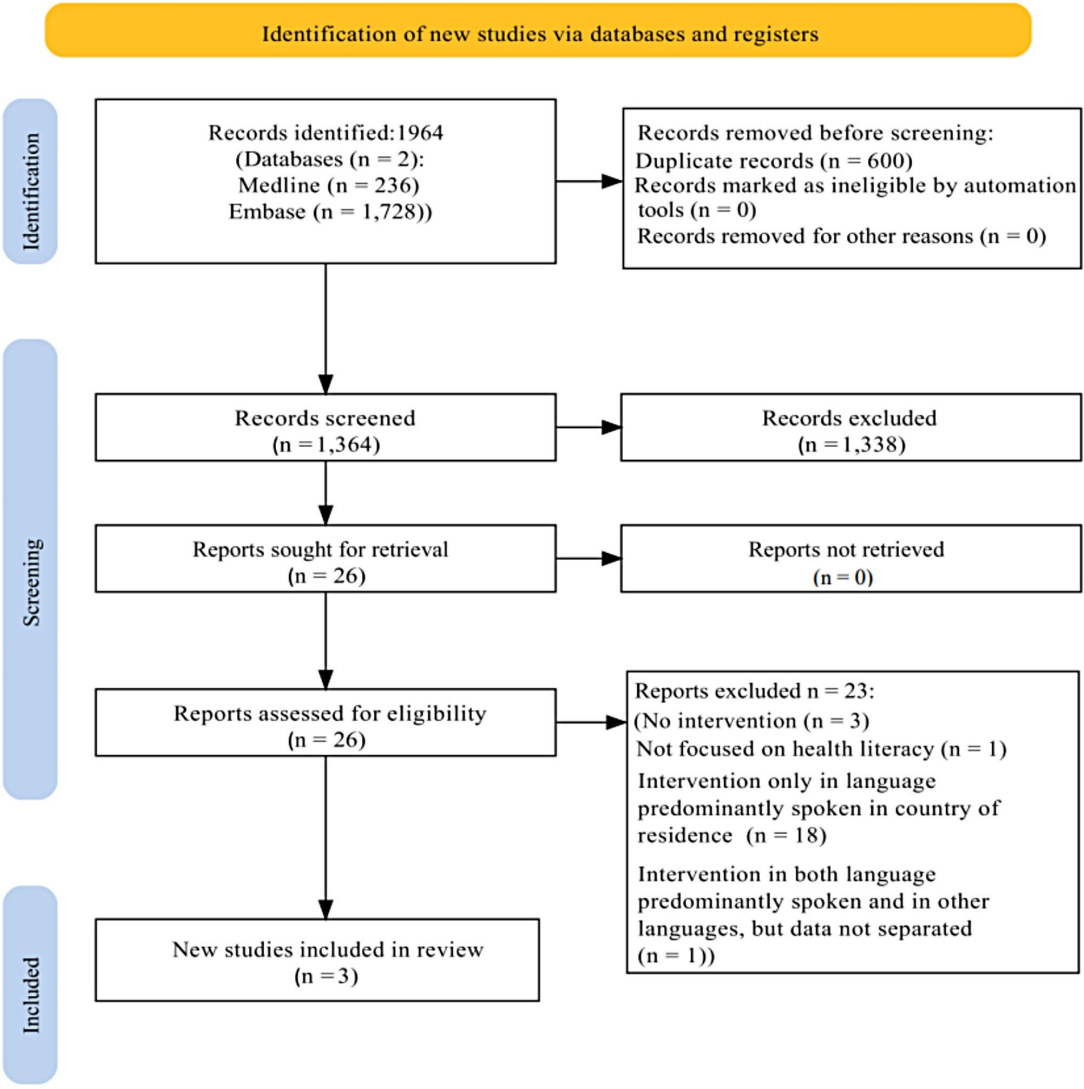
PRISMA flow chart of study selection, inclusion and exclusion

The PRISMA flow chart Fig. 1 illustrates the search process and outcome. The database search resulted in 1964 articles. After duplicates were removed 1364 remained. Titles and abstracts were screened in accordance with the pre-specified inclusion and exclusion criteria. In total, 1338 articles did not meet the eligibility criteria. Full text articles from the remaining 26 abstracts were retrieved. Full texts were assessed and after screening, 3 articles were identified for inclusion in this review. Due to the heterogeneity of the interventions identified and of the outcome measures, with one of the studies not presenting any measured outcomes at all, a meta-analysis of the data was not possible.

### Quality assessment

The MMAT quality assessment tool was used to grade evidence [55]. All three of the included studies - Rasmussen et al. [57], Bartlett et al. [58], and Dougherty et al. [59] - were graded as moderate level evidence. A more detailed presentation of how each MMAT criterion was rated has been summarised in a table which can be found as supplementary material - see Supplementary Table 1, Additional file 1.

### Characteristics of included studies

Included studies ranged in design and included: a cluster randomised controlled trial [57], mixed methods design [59]; and qualitative design [58]. Two studies were carried out in Australia [58, 59], while the third study was undertaken in Denmark [57]. All studies were conducted within the last four years (from 2020 to 2023). A summary table of the included studies can be found as supplementary material - see Supplementary Table 2, Additional file 2.

### Type of health literacy intervention

Studies included distinct interventions aimed at enhancing health literacy among women during different reproductive periods. Rasmussen et al. [57] present the ‘MAMAACT intervention’, a complex health literacy initiative which targeted communication between midwives and immigrant pregnant women of non-Western origin during antenatal care visits in Denmark. The intervention aimed to reduce ethnic and social disparities in stillbirth and infant death by improving communication between pregnant women and midwives regarding warning signs of pregnancy complications. The intervention targets the enhancement of ‘active engagement’ and ‘navigating the healthcare system’ domains of health literacy, with an additional goal of improving pregnant women’s responses to complication signs. This intervention took the form of a six-hour training session and two follow-up dialogue sessions for midwives, as well as a smartphone application and leaflet for the pregnant women in six different languages – Danish, English Arabic, Turkish, Somali, and Urdu.

Bartlett et al. [58] present a health literacy intervention which focused on preconception, pregnancy, and postnatal care, developed for Arabic- and Dari- speaking women of refugee and migrant backgrounds living in Australia, with the aim to improve their maternal health literacy. There was no mention of any specific health literacy domains of focus. The study describes the process of developing the intervention using a design thinking methodology. Stages contributing to this process include the development of user journey maps and posters, user testing of the posters as facilitated in-person by bicultural health educators (BHEs), the development of scripts from the posters, and the subsequent development and user testing of videos. Progression through these stages incorporated feedback and iterative contributions from health care staff, key support networks and women who spoke Arabic or Dari as a first language, who had previously used maternity services in Australia. Five posters and four animated educational videos sharing information on preconception care, early pregnancy support, antenatal care, labour and birth, and postnatal care were the resultant intervention developed.

The intervention presented in the Dougherty et al. [59] article was targeted at migrant women in the postpartum period. The target population was specifically migrant Bangla-speaking Bangladeshi and Mandarin-speaking Chinese new mothers and grandmothers with a baby aged 0 to 1 year (though grandmothers were not relevant to our inclusion criteria for this review). Health literacy domains targeted with this intervention were knowledge, access to information and social support. The intervention was in the form of culturally redesigned new parent classes, including content identified via a co-design workshop with Child and Family Health Nurses (CFHNs) and Bilingual Community Researchers (BCRs). The four session topics included: adjusting to motherhood, childhood illness, transitions during parenthood, and where to go for information and in what circumstances. They were delivered as 2-hour sessions once a week over a 4-week period by CFHNs with the help of interpreters and BCRs.

### Methods used to evaluate intervention effectiveness

In accordance with our secondary aims, we present description of the methods used to evaluate intervention effectiveness of the included studies. Two of the three included studies assessed intervention effectiveness [57, 59]. Rasmussen et al. [57] employed the ‘MAMAACT survey’ to evaluate the effectiveness of their intervention. Though this primarily focused on items from the validated Health Literacy Questionnaire (HLQ), the survey also consisted of questions related to pregnancy, knowledge of handling warning signs of pregnancy complications, satisfaction with midwife antenatal visits, use of interpreters, and socio-demographic characteristics. The surveys were conducted through telephone interview completed in one of the six languages by bilingual and trained interviewers. The focus was on two key domains of the HLQ: ‘active engagement’ and ‘navigating the healthcare system.’ The ‘active engagement’ domain gauged participants’ ease or difficulty in communicating with healthcare providers and discussing health concerns. In parallel, the ‘navigating the healthcare system’ domain assessed the ease or difficulty participants faced in finding the right healthcare, accessing necessary healthcare providers, and understanding healthcare services. Additionally, the incorporation of a purpose-designed question concerning the ‘certainty of complication management’, involved assessing pregnant women’s confidence in handling specific situations related to pregnancy complications, specifically sudden swelling, severe headache, and vaginal bleeding. Intervention effects were analysed for both the total study sample, and in a subsample of women with a non-Western immigrant background. Qualitative analyses were also carried out on in-depth interviews of non-Western immigrant women’s attitudes and experiences with the intervention. This comprehensive approach allowed for a thorough evaluation of the complex intervention.

Similarly, Dougherty et al. [59] used a combination of qualitative and quantitative data collection for process and outcome evaluation of their intervention. The same validated HLQ that was used by Rasmussen et al. [57] was also used in this study pre- and post-intervention, but exploring different domains: domain 2 ‘having sufficient information to manage health’, domain 4 ‘social support for health’, and domain 8 ‘ability to find good health information’. Beyond health literacy levels, further evaluation of this intervention included exploration of recruitment and attendance of participants, feasibility of the intervention, and provider understanding of barriers to health care access. These were measured via focus groups conducted with an interpreter and conversation guide, and semi-structured interviews with the BCRs and CFHNs, assisted by an interview guide. Bartlett et al. [58] described plans to evaluate the effectiveness of the animated videos, though, no publication demonstrating an evaluation of their health literacy intervention was identified at the time of article retrieval for this review.

### Outcomes of the health literacy interventions

Outcomes of the identified health literacy interventions were varied. Two of the three included studies, Rasmussen et al. [57] and Dougherty et al. [59] reported on outcomes of their health literacy programmes. Rasmussen et al. [57] reported findings whereby the intervention did not show an enhancement in the health literacy of pregnant women concerning active engagement with healthcare providers and navigating the healthcare system. This was the case for the total study sample, as well as in the subgroup analysis of women with a non-Western immigrant background. Nevertheless, women who underwent the intervention exhibited a greater certainty in knowing how to respond to warning signs of pregnancy complications when compared to their counterparts in the control group. Dougherty et al. [59] analysed data for those who attended three or more of the four intervention sessions and found that both the Bangla- and Mandarin-speaking groups had higher health literacy scores across all three health literacy domains at the post-intervention survey. Both groups’ health literacy scores were also compared with the average scores from (1) a sample of Australians aged above 18 and (2) a sample of Australians who speak a language other than English (LOTE) at home, as surveyed by the Australian Bureau of Statistics (ABS). For domain 2, both the Bangla- and Mandarin- speaking groups equalled or exceeded both ABS averages at the post-intervention time point. For domain 4, both groups equalled or exceeded the average score from the LOTE ABS sample at the post-intervention time point. Lastly, for domain 8, both groups were close to but remained slight below the average ABS scores for both the Australian sample aged over 18 and the LOTE sample. Thus, this study was able to demonstrate the effectiveness of the culturally redesigned classes as an intervention in improving these three health literacy domains [59].

## Discussion

Health literacy is paramount in pregnancy. It has been well established that low health literacy in pregnancy is associated with poorer maternal and infant health outcomes. Considering women have a substantially increased frequency of contact with healthcare professionals during this time, the pregnancy period represents a pivotal opportunity to improve health literacy [12, 13]. Migrant populations have generally been shown to have lower levels of health literacy compared to the native population [38–40], particularly if they lack proficiency in the language of the health care system [45].

There has been an evident trend in increasing international migration over time [60], with more women experiencing pregnancy and childbirth in countries where the main language spoken is different from their first language. In response to global maternal populations continuing to diversify, healthcare providers, governing bodies, and policymakers must not only adapt services to cater to these populations with varying language abilities, but also help enable them to become more autonomous in effectively navigating healthcare systems in pregnancy. Extending support beyond current conventional healthcare to include health literacy, would empower these pregnant women to take active roles in their own health management.

There is published literature reviewing health literacy levels, associations with health literacy [14] and the effectiveness of health literacy interventions in general pregnant populations [35]. However, this appears to be the first ever systematic review conducted which focuses on identifying health literacy interventions, targeted specifically to pregnant women whose first language is one other than the language predominantly spoken in their country of residence.

Our review led to the identification of three interventions. All three intervention modalities differed significantly, with one involving midwife training sessions combined with a smartphone application and leaflet for pregnant women [57], another involving posters and animated educational videos [58], and the last in the form of culturally adapted new parent classes [59]. A previous review of health literacy interventions in general pregnant populations presented their identified studies as classified by intervention design; the groups were ‘decision-aid interventions’, ‘face-to-face interventions’ and ‘written interventions’ [35]. Statistically significant improvements in knowledge were found in studies across each of these different groups [35]. Thus, both the previous review [35] and our review highlight the wide scope of modalities which can be utilised to develop effective health literacy interventions in pregnancy. In our review, two of the three identified interventions evaluated intervention effectiveness. The culturally adapted new parent classes showed an improvement in the health literacy domains being measured [59], whilst the other (‘MAMAACT intervention’) did not [57]. The latter intervention was, however, associated with greater certainty in knowing how to respond to warning signs of pregnancy complications [57].

We noted some key considerations which may need to be given in developing health literacy interventions catered specifically to pregnant women whose first language differs to the one predominantly spoken in the country they live in. Two of the three identified studies aimed to use co-design with community representatives [58, 59], but due to Coronavirus disease (COVID-19) lockdown restrictions limiting the number of community feedback sessions, one of these studies resorted to user testing as part of a design thinking methodology instead [58]. Using these collaborative design techniques in the development of health literacy interventions appears to be integral in ensuring the content and delivery of these interventions are culturally sensitive and meet the unique needs of each diverse target population.

We also recognised the invaluable and crucial involvement of interpreter services, BHEs [58] and of BCRs [59]. Various translating services were used for multiple rounds of both forward and reverse translation of the intervention content, to ensure accuracy. BHEs helped facilitate in-person user testing sessions with the participants, using prompts to elicit their feedback on the health literacy intervention, and assisting in translating these responses back to the authors [58]. BCRs were defined as ‘an emerging workforce who provide research support in languages other than English’ [59]. They helped with the development of the intervention, recruiting new mothers to the intervention, and providing cultural support during delivery of the intervention. In both the Mandarin- and Bangla- speaking groups, attendance was greater for participants recruited by BCRs as compared to those recruited by the CFHNs. Dougherty et al. felt that the BCRs role was a key contributor to the success of the health literacy intervention, as their shared language and cultural identity resulted in connections and trust within the participants’ communities [59].

A further important consideration is that the health literacy tools should be translated to different languages via a robust method, to enable outcome assessment in linguistically diverse cohorts. Although the HLQ has been validated in many different languages, it had not been validated in Urdu [57] and Bangla [59] when used in our included articles. The authors of one article translated the HLQ to Urdu according to the Health Literacy Questionnaire Integrity Procedure to ensure accurate translation [57].

A recent systematic realist review found that second-language courses (SLCs) for migrants had a positive effect on different components of health literacy [61]. Harsch et al. subsequently developed a theory of change stating that SLCs / language training courses are complex interventions which can promote migrants’ health literacy [61]. Thus, in future efforts to improve health literacy in migrant populations, it appears beneficial to also incorporate some level of teaching of the language predominantly spoken in their country of residence, alongside interventions translated to their own first language.

In addition to inadequate health literacy, other barriers to prenatal care utilisation found in an immigrant Latino population included unauthorised immigration status leading to low insurance cover, fear of deportation, difficult and lengthy forms to apply for healthcare coverage, inadequate interpretation services, issues with transportation access and mental health distress [62]. It would be invaluable to consider concomitant barriers and contexts when developing interventions for women from these demographics, to enable more holistic, targeted support. We suggest that education and support with regards to these other barriers, should also be integrated into future health literacy interventions.

International public health authorities such as the World Health Organization (WHO) have specifically emphasised the importance of and recommended the use of health literacy initiatives as a health promotion strategy for migrants, as well as refugees [63]. We propose that funding should be set aside for the development of health literacy interventions and research targeting these groups of pregnant women that have limited language proficiency, including their active involvement in these processes [64]. There is also an opportunity to recognise the broader implications of empowering this cohort of pregnant women through improved health literacy acquisition; to both facilitate better health outcomes, but also to improve confidence, employability [65], and socio-economic status [66]. Adopting such an approach could create ripple effects that extend benefits to children and families through generations, contributing to a healthier more equitable society.

### Strengths and limitations

The review was conducted in accordance with PRISMA guidelines; key strengths include the blinding of all reviewers, with the independent assessment of studies for eligibility and for quality appraisal. A key limitation we recognise in our work is that we did not search all existing databases, including the grey literature. Despite this, we used Embase, which is the largest database on Ovid. Furthermore, grey literature lacks rigorous peer-review and thus consists of lower quality studies. Therefore, we excluded grey literature to ensure a higher standard of evidence in our systematic review. Nonetheless, we acknowledge that this increases the risk of publication bias. Another limitation is that the search terms we used may have been too narrow. Had we also used the term ‘patient education’ for an example, we may have captured studies with interventions aimed to improve health literacy, though they do not explicitly mention the term ‘health literacy’. We also only included articles published in English. Hence, there is potential that we have missed relevant literature. The intervention types and outcome measures for the studies identified in this review were all different. Despite two of the identified studies both using the same HLQ as a part of their outcome measures, each focused on different domains of the questionnaire; the third identified study did not present any measured outcomes at all. As a result, a meta-analysis was not possible due to these inconsistencies.

## Conclusions

Our review found a scarce number of health literacy interventions for pregnant women whose first language differs from the language predominantly spoken in the country they live in. The diverse nature of the interventions identified highlights the potential for employing a range of strategies to improve health literacy and address disparities in maternal and infant outcomes. The variability in the effectiveness of these interventions underscores the importance of developing tailored approaches that consider the specific needs, contexts, and challenges faced by this cohort. Future efforts should focus on developing, trialling, and evaluating such interventions, with the active involvement of the pregnant women who lack proficiency in the predominant language of their residing country, and those who support them, to ensure their relevance and effectiveness. Bridging the health literacy gap for linguistically diverse pregnant populations presents an opportunity to advance equitable healthcare outcomes for pregnant women and their children.

## Electronic supplementary material

Below is the link to the electronic supplementary material.

Supplementary Material 1: Mixed Methods Appraisal Tool (MMAT) [55] quality assessment of included studies - a more detailed presentation of how each MMAT criterion was rated.

Supplementary Material 2: Summary table of results-table containing a breakdown of each included study.

## Data Availability

No datasets were generated or analysed during the current study.

## Abbreviations

PRISMA: Preferred Reporting Items for Systematic Reviews and Meta-analyses
PICOS: Population, Intervention, Comparison, Outcome, Study design
MMAT: Mixed Methods Appraisal Tool
BHE: Bicultural Health Educators
CFHNs: Child and Family Health Nurses
BCRs: Bilingual Community Researchers
HLQ: Health Literacy Questionnaire
LOTE: Language Other Than English
ABS: Australian Bureau of Statistics
COVID-19: Coronavirus disease
SLCs: Second-Language Courses
WHO: World Health Organization

## References

[1] Dodson S, Good S, Osborne RH. Optimizing health literacy: improving health and reducing health inequities: a selection of information sheets from the health literacy toolkit for low- and middle- income countries. World Health Organisation, Regional Office for South-East Asia; 2015. ISBN: 978-92-9022-474-7.

[2] Parnell TA. 1: health literacy: history, definitions, and models. Health literacy in nursing. Springer Publishing; 2014. pp. 3–31.

[3] Sørensen K, Van den Broucke S, Fullam J, Doyle G, Pelikan J, Slonska Z, et al. Health literacy and public health: a systematic review and integration of definitions and models. BMC Public Health. 2012;12:80. 22276600 10.1186/1471-2458-12-80 PMC3292515

[4] Liu C, Wang D, Liu C, Jiang J, Wang X, Chen H, et al. What is the meaning of health literacy? A systematic review and qualitative synthesis. Family Med Community Health. 2020;8(2):e000351. 10.1136/fmch-2020-000351 PMC7239702 32414834

[5] Santana S, Brach C, Harris L, Ochiai E, Blakey C, Bevington F, et al. Updating health literacy for healthy people 2030: defining its importance for a new decade in public health. J Public Health Manage Pract. 2021;27(Supplement 6):pS258–S264. 10.1097/PHH.0000000000001324 PMC8435055 33729194

[6] Berkman ND, Sheridan SL, Donahue KE, Halpern DJ, Crotty K. Low health literacy and health outcomes: an updated systematic review. Ann Intern Med. 2011;155(2):97–107. 21768583 10.7326/0003-4819-155-2-201107190-00005

[7] Mantwill S, Diviani N. Health literacy and health disparities: A global perspective. In Okan O, Bauer U, Levin-Zamir D, Pinheiro P, Sørensen K, editors, International handbook of health literacy: Research, practice and policy across the life-span; 2019. pp. 139–152.

[8] Stormacq C, Van den Broucke S, Wosinski J. 2019. Does health literacy mediate the relationship between socioeconomic status and health disparities? Integrative review. Health Promot. Int. 2019;34(5):e1–17. 10.1093/heapro/day062 30107564

[9] Rezaee R, Ravangard R, Amani F, Dehghani Tafti A, Shokrpour N, Bahrami MA. Healthy lifestyle during pregnancy: uncovering the role of online health information seeking experience. PLoS ONE. 2022;17(8). 10.1371/journal.pone.0271989 PMC9342740 35913949

[10] Hay SJ, McLachlan HL, Newton M, Forster DA, Shafiei T. Sources of information during pregnancy and the early parenting period: exploring the views of women and their partners. Midwifery. 2022;105:103236. 34968821 10.1016/j.midw.2021.103236

[11] Olander EK, Smith DM, Darwin Z. Health behaviour and pregnancy: a time for change. J Reproductive Infant Psychol. 2018;36(1):1–3. 10.1080/02646838.2018.1408965 29517295

[12] Locke A. Putting the ‘teachable moment’ in context: a view from critical health psychology. J Health Psychol. 2023;28(1):3–16. 35672937 10.1177/13591053221101750 PMC9909030

[13] Yee LM, Simon MA, Grobman WA, Rajan PV. Pregnancy as a golden opportunity for patient activation and engagement. Am J Obstet Gynecol. 2021;224(1):116–8. 32979375 10.1016/j.ajog.2020.09.024

[14] Nawabi F, Krebs F, Vennedey V, Shukri A, Lorenz L, Stock S. Health Literacy in pregnant women: a systematic review. Int J Environ Res Public Health. 2021;18(7):3847. 33917631 10.3390/ijerph18073847 PMC8038834

[15] Lupattelli A, Picinardi M, Einarson A, Nordeng H. Health literacy and its association with perception of teratogenic risks and health behaviour during pregnancy. Patient Educ Couns. 2014;96(2):171–8. 24862909 10.1016/j.pec.2014.04.014

[16] Sahin E, Yesilcinar I, Geris R, Pasalak SI, Seven M. The impact of pregnant women’s health literacy on their health-promoting lifestyle and teratogenic risk perception. Health Care Women Int. 2021;42(4–6):598–610. 32744890 10.1080/07399332.2020.1797036

[17] Van Schendel RV, Page-Christiaens GC, Beulen L, Bilardo CM, de Boer MA, Coumans AB, et al. Trial by Dutch Laboratories for evaluation of non-invasive prenatal testing. Part II – women’s perspectives. Prenat Diagn. 2016;36:1091–8. 27739584 10.1002/pd.4941 PMC5213994

[18] Sheinis M, Carpe N, Gold S, Selk A. Ignorance is bliss: women’s knowledge regarding age-related pregnancy risks. J Obstet Gynaecol. 2018;38(3):344–51. 29022426 10.1080/01443615.2017.1357685

[19] Valero-Chillerón MJ, Mena-Tudela D, Cervera-Gasch Á, González-Chordá VM, Soriano-Vidal FJ, Quesada JA, et al. Influence of Health Literacy on Maintenance of Exclusive Breastfeeding at 6 months Postpartum: a Multicentre Study. Int J Environ Res Public Health. 2022;19(9):5411. 35564807 10.3390/ijerph19095411 PMC9104596

[20] Stafford JD, Goggins ER, Lathrop E, Haddad LB. Health Literacy and Associated Outcomes in the Postpartum period at Grady Memorial Hospital. Matern Child Health J. 2021;25(4):599–605. 33196925 10.1007/s10995-020-03030-1

[21] Tsai TI, Huang SH, Lee SYD. Maternal and Hospital Factors Associated with First-Time mothers’ breastfeeding practice: a prospective study. Breastfeed Med. 2015;10(6):334–40. 26110594 10.1089/bfm.2015.0005

[22] Guler DS, Sahin S, Ozdemir K, Unsal A, Uslu Yuvacı H. Health literacy and knowledge of antenatal care among pregnant women. Health Soc Care Community. 2021;29(6):1815–23. 33484046 10.1111/hsc.13291

[23] Brandstetter S, Rothfuß D, Seelbach-Göbel B, Melter M, Kabesch M, Apfelbacher C et al. Information on, knowledge and utilisation of support services during pregnancy and after childbirth: cross-sectional analyses of predictors using data from the KUNO-Kids health study. BMJ Open. 2020;10(10). 10.1136/bmjopen-2020-037745 PMC7592309 33109648

[24] Probandari A, Arcita A, Kothijah K, Pamungkasari EP. Barriers to utilization of postnatal care at village level in Klaten District, central Java Province, Indonesia. BMC Health Serv Res. 2017;17(1):541. 28784169 10.1186/s12913-017-2490-y PMC5547562

[25] Bello CB, Esan DT, Akerele SA, Fadare RI. Maternal health literacy, utilisation of maternal healthcare services and pregnancy outcomes among newly delivered mothers: a cross-sectional study in Nigeria. Public Health Pract. 2022;3:100266. 10.1016/j.puhip.2022.100266 PMC9461586 36101756

[26] Putra DA, Prihatanto FSI, Lestari P. Health Literacy and Pre-eclampsia Knowledge of Pregnant Mother in Primary Health Care in Surabaya. Biomol Health Sci J. 2020;3(2):81–3.

[27] Santi DR, Suminar DR, Devy SR, Mahmudah M. Health Literacy and Pregnant Women Behaviors in early detection of Preeclampsia warning signs: Cross Sectional Study in Rural areas of Indonesia. NeuroQuantology. 2022;20(10):9450–8.

[28] Eser N, Çelik N. Association between rational drug use and health literacy among pregnant women: a cross-sectional study. Women Health. 2022;62(7):612–20. 35861057 10.1080/03630242.2022.2100033

[29] Smedberg J, Lupattelli A, Mårdby AC, Nordeng H. Characteristics of women who continue smoking during pregnancy: a cross-sectional study of pregnant women and new mothers in 15 European countries. BMC Pregnancy Childbirth. 2014;14:213. 24964728 10.1186/1471-2393-14-213 PMC4080751

[30] Vila-Candel R, Navarro-Illana E, Mena-Tudela D, Pérez-Ros P, Castro-Sánchez E, Soriano-Vidal FJ, et al. Influence of Puerperal Health Literacy on Tobacco Use during pregnancy among Spanish women: a transversal study. Int J Environ Res Public Health. 2020;17(8):2910. 32340128 10.3390/ijerph17082910 PMC7216153

[31] Shieh C, Mays R, McDaniel A, Yu J. Health literacy and its association with the use of information sources and with barriers to information seeking in clinic-based pregnant women. Health Care Women Int. 2009;30(11):971–88. 19809901 10.1080/07399330903052152

[32] Endres LK, Sharp LK, Haney E, Dooley SL. Health Literacy and Pregnancy Preparedness in Pregestational Diabetes. Diabetes Care. 2004;27(2):331–4. 14747209 10.2337/diacare.27.2.331

[33] Pirdehghan A, Eslahchi M, Esna-Ashari F, Borzouei S. Health literacy and diabetes control in pregnant women. J Family Med Prim Care. 2020;9(2):1048–52. 32318465 10.4103/jfmpc.jfmpc_891_19 PMC7114019

[34] Yee LM, Silver R, Haas DM, Parry S, Mercer BM, Wing DA, et al. Association of Health Literacy among Nulliparous Individuals and maternal and neonatal outcomes. JAMA Netw Open. 2021;4(9):e2122576. 34468757 10.1001/jamanetworkopen.2021.22576 PMC8411280

[35] Zibellini J, Muscat DM, Kizirian N, Gordon A. Effect of health literacy interventions on pregnancy outcomes: a systematic review. Women Birth. 2021;34(2):180–6. 32094036 10.1016/j.wombi.2020.01.010

[36] Thapa-Bajgain K, Bajgain BB, Dahal R, Adhikari K, Chowdhury N, Chowdhury MZ, et al. Health literacy among members of the Nepalese immigrant population in Canada. Health Educ J. 2023;82(3):274–85.

[37] Medina P, Maia AC, Costa A. Health Literacy and Migrant Communities in Primary Health Care. Front Public Health. 2022;9:798222. 35141189 10.3389/fpubh.2021.798222 PMC8818741

[38] Bergman L, Nilsson U, Dahlberg K, Jaensson M, Wangdahl J. Health literacy and e-health literacy among arabic-speaking migrants in Sweden: a cross-sectional study. BMC Public Health. 2021;21(2165). 10.1186/s12889-021-12187-5 PMC8614220 34823499

[39] Villadsen SF, Hadi H, Ismail I, Osborne RH, Ekstrøm CT, Kayser L. Ehealth literacy and health literacy among immigrants and their descendants compared with women of Danish origin: a cross-sectional study using a multidimensional approach among pregnant women. BMJ Open. 2020;10(5). 10.1136/bmjopen-2020-037076 PMC7228522 32385065

[40] Brorsen E, Rasmussen TD, Ekstrøm CT, Osborne RH, Villadsen SF. Health literacy responsiveness: a cross-sectional study among pregnant women in Denmark. Scand J Public Health. 2022;50(4):507–15. 33863261 10.1177/14034948211004320

[41] Hickey KT, Masterson Creber RM, Reading M, Sciacca RR, Riga TC, Frulla AP, et al. Low health literacy: implications for managing cardiac patients in practice. Nurse Pract. 2018;43(8):49–55. 30028773 10.1097/01.NPR.0000541468.54290.49 PMC6391993

[42] Sentell T, Braun KL. Low health literacy, limited English proficiency, and health status in asians, latinos, and other racial/ethnic groups in California. J Health Commun. 2012;17(Suppl 3):82–99. 23030563 10.1080/10810730.2012.712621 PMC3552496

[43] Singh H, Kolschen J, Samkange-Zeeb F, Brand T, Zeeb H, Schuz B. Modifiable predictors of health literacy in working-age adults - a rapid review and meta-analysis. BMC Public Health. 2022;22(1450). 10.1186/s12889-022-13851-0 PMC9338662 35906567

[44] Fernandez Reino M. English language use and proficiency of migrants in the UK, Migration Observatory briefing. COMPAS, University of Oxford; 2019.

[45] Becerra BJ, Arias D, Becerra MB. Low Health Literacy among immigrant hispanics. J Racial Ethnic Health Disparities. 2017;4(3):480–3. 10.1007/s40615-016-0249-5 27324821

[46] Bains S, Sundby J, Lindskog BV, Vangen S, Sørbye IK. Newly arrived migrant women’s experience of Maternity Health Information: a face-to-face Questionnaire Study in Norway. Int J Environ Res Public Health. 2021;18(14):7523. 34299974 10.3390/ijerph18147523 PMC8307311

[47] Almeida LM, Caldas J, Ayres-de-Campos D, Salcedo-Barrientos D, Dias S. Maternal healthcare in migrants: a systematic review. Matern Child Health J. 2013;17(8):1346–54. 23334357 10.1007/s10995-012-1149-x

[48] Behboudi-Gandevani S, Bidhendi-Yarandi R, Panahi MH, Mardani A, Paal P, Prinds C, et al. Adverse pregnancy outcomes and International Immigration Status: a systematic review and Meta-analysis. Ann Glob Health. 2022;88(1):44. 35854922 10.5334/aogh.3591 PMC9248985

[49] Togioka BM, Seligman KM, Delgado CM. Limited English proficiency in the labor and delivery unit. Curr Opin Anaesthesiol. 2022;35(3):285–91. 35671014 10.1097/ACO.0000000000001131

[50] Sentell T, Chang A, Ahn HJ, Miyamura J. Maternal language and adverse birth outcomes in a statewide analysis. Women Health. 2016;56(3):257–80. 26361937 10.1080/03630242.2015.1088114 PMC4868388

[51] Hughson J, Marshall F, Daly JO, Woodward-Kron R, Hajek J, Story D. Health professionals’ views on health literacy issues for culturally and linguistically diverse women in maternity care: barriers, enablers and the need for an integrated approach. Aust Health Rev. 2017;42(1):10–20. 10.1071/AH17067 29081348

[52] Page MJ, McKenzie JE, Bossuyt PM, Boutron I, Hoffman TC, Mulrow CD, et al. The PRISMA 2020 statement: an updated guideline for reporting systematic reviews. Syst Rev. 2021;10(1):89. 33781348 10.1186/s13643-021-01626-4 PMC8008539

[53] Amir-Behghadami M, Janati A, Population. Intervention, comparison, outcomes and study (PICOS) design as a framework to formulate eligibility criteria in systematic reviews. Emerg Med J. 2020;37(6):387. 32253195 10.1136/emermed-2020-209567

[54] Smith PG, Morrow RH, Ross DA, editors. Chapter 2, Types of intervention and their development. Field Trials of Health Interventions: A Toolbox. 3rd edition. Oxford (UK): OUP Oxford. 2015; 10.1093/med/9780198732860.001.0001 26225404

[55] Hong QN, Pluye P, Fàbregues S, Bartlett G, Boardman F, Cargo M et al. Mixed methods appraisal tool (MMAT), version 2018 user guide. McGill University Department of Family Medicine; 2018. http://mixedmethodsappraisaltoolpublic.pbworks.com/w/file/fetch/127916259/MMAT_2018_criteria-manual_2018-08-01_ENG.pdf

[56] Popay J, Roberts H, Sowden A, Petticrew M, Arai L, Rodgers M et al. Guidance on the conduct of narrative synthesis in systematic reviews: a product from the ESRC Methods Programme. 2006; 10.13140/2.1.1018.4643

[57] Rasmussen TD, Andersen AN, Ekstrom CT, Jervelund SS, Villadsen SF. Improving health literacy responsiveness to reduce ethnic and social disparity in stillbirth and infant health: a cluster randomized controlled effectiveness trial of the MAMAACT intervention. Int J Nurs Stud. 2023;144:104505. 37267853 10.1016/j.ijnurstu.2023.104505

[58] Bartlett R, Boyle JA. Developing multi-language maternal health education videos for refugee and migrant women in southeast Melbourne. Midwifery. 2022;111:103369. 35617881 10.1016/j.midw.2022.103369

[59] Dougherty L, Riley A, Caffrey P, Wallbank A, Milne M, Harris MF, et al. Supporting newly arrived migrant mothers: a Pilot Health literacy intervention. Health Lit Res Pract. 2021;5(3):e201–7. 34260320 10.3928/24748307-20210601-01 PMC8280910

[60] Mcauliffe M, Triandafylliidou A. World migration report 2022. International Organisation for Migration (IOM); 2021. ISBN: 978-92-9268-078-7.

[61] Harsch S, Bittlingmayer U. Advancing the health literacy of migrants in second-language courses: Realist review. Int Health Trends Perspect. 2024;4(1):40–67.

[62] Camargo JT, Barral RL, Kerling EH, Saavedra L, Carlson SE, Gajewski BJ, et al. Prenatal care utilization challenges and facilitators for a growing latino community in the Midwest. Matern Child Health J. 2023;27:1811–22. 37369811 10.1007/s10995-023-03733-1 PMC11251489

[63] World Health Organization. Regional Office for Europe. Health promotion for improved refugee and migrant health: technical guidance. 2018. https://iris.who.int/handle/10665/342287. Accessed 12 April 2024.

[64] Moss N, Daru J, Lanz D, Thangaratinam S, Khan KS. Involving pregnant women, mothers and members of the public to improve the quality of women’s health research. BJOG. 2017;124:362–5. 27862921 10.1111/1471-0528.14419

[65] Ehmann AT, Ög E, Rieger MA, Siegel A. Work-related health literacy: a scoping review to clarify the Concept. Int J Environ Res Public Health. 2021;18(19):9945. 34639262 10.3390/ijerph18199945 PMC8507793

[66] Svendsen MT, Bak CK, Sørensen K, Pelikan J, Riddersholm SJ, Skals RK, et al. Associations of health literacy with socioeconomic position, health risk behavior, and health status: a large national population-based survey among Danish adults. BMC Public Health. 2020;20:565. 32345275 10.1186/s12889-020-08498-8 PMC7187482

