# Supplementary material for: Health literacy interventions for pregnant women with limited language proficiency in the country they live in: a systematic review
Source: BMC Public Health. 2024 Nov 26;24:3287. doi: 10.1186/s12889-024-20747-8 (PMC11600627; doi:10.1186/s12889-024-20747-8)
Supplement: Supplementary file 1 — Supplementary Material 1: Mixed Methods Appraisal Tool (MMAT) [55] quality assessment of included studies - a more detailed presentation of how each MMAT criterion was rated. [file 12889_2024_20747_MOESM1_ESM.docx]

Supplementary Table 1, Additional file 1. Mixed Methods Appraisal Tool (MMAT) [55] quality assessment of included studies

1. Rasmussen et al. [57] - Cluster randomised controlled trial

| **Mixed Methods Appraisal Tool (MMAT) Version 2018** | | | | | |
| --- | --- | --- | --- | --- | --- |
| **Category of study design** | **Methodological quality criteria** | **Responses** | | | |
|  |  | Yes | No | Can’t tell | Comments |
| Screening questions (for all types) | S1. Are there clear research questions? | Yes |  |  |  |
|  | S2. Do the collected data allow to address the research questions? | Yes |  |  |  |
|  | *Further appraisal may not be feasible when the answer is ‘No’ or ‘Can’t tell’ to one or both screening questions* | | | | |
| 1. Qualitative | 1.1 Is the qualitative approach appropriate to answer the research question? |  |  |  |  |
|  | 1.2. Are the qualitative data collection methods adequate to address the research question? |  |  |  |  |
|  | 1.3. Are the findings adequately derived from the data? |  |  |  |  |
|  | 1.4. Is the interpretation of results sufficiently substantiated by data? |  |  |  |  |
|  | 1.5. Is there coherence between qualitative data sources, collection, analysis and interpretation? |  |  |  |  |
| 1. Quantitative randomised controlled trials | 2.1. Is randomization appropriately performed? | Yes |  |  |  |
|  | 2.2. Are the groups comparable at baseline? | Yes |  |  |  |
|  | 2.3. Are there complete outcome data? | No |  |  | - Our agreed cut-off value for acceptable complete outcome data was 80% |
|  | 2.4. Are outcome assessors blinded to the intervention provided? | No |  |  |  |
|  | 2.5 Did the participants adhere to the assigned intervention? | No |  |  | - Some women changed study arm during study period (e.g. due to moving / complications requiring specialist antenatal care at another hospital). However, sensitivity analyses excluding women who changed study arm did not change the overall results - Contamination of the intervention across intervention and control groups was indicated in the sub-group of women with a non-Western immigrant background (screenshots of MAMAACT app shared with friends in control group) - Intention-to-treat analysis performed |
| 1. Quantitative non-randomised | 3.1. Are the participants representative of the target population? |  |  |  |  |
|  | 3.2. Are measurements appropriate regarding both the outcome and intervention (or exposure)? |  |  |  |  |
|  | 3.3. Are there complete outcome data? |  |  |  |  |
|  | 3.4. Are the confounders accounted for in the design and analysis? |  |  |  |  |
|  | 3.5. During the study period, is the intervention administered (or exposure occurred) as intended? |  |  |  |  |
| 1. Quantitative descriptive | 4.1. Is the sampling strategy relevant to address the research question? |  |  |  |  |
|  | 4.2. Is the sample representative of the target population? |  |  |  |  |
|  | 4.3. Are the measurements appropriate? |  |  |  |  |
|  | 4.4. Is the risk of nonresponse bias low? |  |  |  |  |
|  | 4.5. Is the statistical analysis appropriate to answer the research question? |  |  |  |  |
| 1. Mixed methods | 5.1. Is there an adequate rationale for using a mixed methods design to address the research question? |  |  |  |  |
|  | 5.2. Are the different components of the study effectively integrated to answer the research question? |  |  |  |  |
|  | 5.3. Are the outputs of the integration of qualitative and quantitative components adequately interpreted? |  |  |  |  |
|  | 5.4. Are divergences and inconsistencies between quantitative and qualitative results adequately addressed? |  |  |  |  |
|  | 5.5. Do the different components of the study adhere to the quality criteria of each tradition of the methods involved? |  |  |  |  |

Rasmussen et al. overall rating: Moderate quality

1. Bartlett et al. [58] - Qualitative

| **Mixed Methods Appraisal Tool (MMAT) Version 2018** | | | | | |
| --- | --- | --- | --- | --- | --- |
| **Category of study design** | **Methodological quality criteria** | **Responses** | | | |
|  |  | Yes | No | Can’t tell | Comments |
| Screening questions (for all types) | S1. Are there clear research questions? | Yes |  |  |  |
|  | S2. Do the collected data allow to address the research questions? | Yes |  |  |  |
|  | *Further appraisal may not be feasible when the answer is ‘No’ or ‘Can’t tell’ to one or both screening questions* | | | | |
| 1. Qualitative | 1.1 Is the qualitative approach appropriate to answer the research question? | Yes |  |  |  |
|  | 1.2. Are the qualitative data collection methods adequate to address the research question? | Yes |  |  |  |
|  | 1.3. Are the findings adequately derived from the data? |  |  | Can’t tell |  |
|  | 1.4. Is the interpretation of results sufficiently substantiated by data? | Yes |  |  |  |
|  | 1.5. Is there coherence between qualitative data sources, collection, analysis and interpretation? |  |  | Can’t tell |  |
| 1. Quantitative randomised controlled trials | 2.1. Is randomization appropriately performed? |  |  |  |  |
|  | 2.2. Are the groups comparable at baseline? |  |  |  |  |
|  | 2.3. Are there complete outcome data? |  |  |  |  |
|  | 2.4. Are outcome assessors blinded to the intervention provided? |  |  |  |  |
|  | 2.5 Did the participants adhere to the assigned intervention? |  |  |  |  |
| 1. Quantitative non-randomised | 3.1. Are the participants representative of the target population? |  |  |  |  |
|  | 3.2. Are measurements appropriate regarding both the outcome and intervention (or exposure)? |  |  |  |  |
|  | 3.3. Are there complete outcome data? |  |  |  |  |
|  | 3.4. Are the confounders accounted for in the design and analysis? |  |  |  |  |
|  | 3.5. During the study period, is the intervention administered (or exposure occurred) as intended? |  |  |  |  |
| 1. Quantitative descriptive | 4.1. Is the sampling strategy relevant to address the research question? |  |  |  |  |
|  | 4.2. Is the sample representative of the target population? |  |  |  |  |
|  | 4.3. Are the measurements appropriate? |  |  |  |  |
|  | 4.4. Is the risk of nonresponse bias low? |  |  |  |  |
|  | 4.5. Is the statistical analysis appropriate to answer the research question? |  |  |  |  |
| 1. Mixed methods | 5.1. Is there an adequate rationale for using a mixed methods design to address the research question? |  |  |  |  |
|  | 5.2. Are the different components of the study effectively integrated to answer the research question? |  |  |  |  |
|  | 5.3. Are the outputs of the integration of qualitative and quantitative components adequately interpreted? |  |  |  |  |
|  | 5.4. Are divergences and inconsistencies between quantitative and qualitative results adequately addressed? |  |  |  |  |
|  | 5.5. Do the different components of the study adhere to the quality criteria of each tradition of the methods involved? |  |  |  |  |

Bartlett et al. overall rating: Moderate quality

3) Dougherty et al. [59] - Mixed methods

| **Mixed Methods Appraisal Tool (MMAT) Version 2018** | | | | | |
| --- | --- | --- | --- | --- | --- |
| **Category of study design** | **Methodological quality criteria** | **Responses** | | | |
|  |  | Yes | No | Can’t tell | Comments |
| Screening questions (for all types) | S1. Are there clear research questions? | Yes |  |  |  |
|  | S2. Do the collected data allow to address the research questions? | Yes |  |  |  |
|  | *Further appraisal may not be feasible when the answer is ‘No’ or ‘Can’t tell’ to one or both screening questions* | | | | |
| 1. Qualitative | 1.1 Is the qualitative approach appropriate to answer the research question? | Yes |  |  | - Qualitative components of mixed methods study appraised |
|  | 1.2. Are the qualitative data collection methods adequate to address the research question? | Yes |  |  |  |
|  | 1.3. Are the findings adequately derived from the data? | Yes |  |  |  |
|  | 1.4. Is the interpretation of results sufficiently substantiated by data? | Yes |  |  |  |
|  | 1.5. Is there coherence between qualitative data sources, collection, analysis and interpretation? | Yes |  |  |  |
| 1. Quantitative randomised controlled trials | 2.1. Is randomization appropriately performed? |  |  |  |  |
|  | 2.2. Are the groups comparable at baseline? |  |  |  |  |
|  | 2.3. Are there complete outcome data? |  |  |  |  |
|  | 2.4. Are outcome assessors blinded to the intervention provided? |  |  |  |  |
|  | 2.5 Did the participants adhere to the assigned intervention? |  |  |  |  |
| 1. Quantitative-non randomised | 3.1. Are the participants representative of the target population? | Yes |  |  | - Quantitative components of mixed methods study appraised |
|  | 3.2. Are measurements appropriate regarding both the outcome and intervention (or exposure)? | Yes |  |  |  |
|  | 3.3. Are there complete outcome data? |  | No |  | - Our agreed cut-off value for acceptable complete outcome data was 80% |
|  | 3.4. Are the confounders accounted for in the design and analysis? |  | No |  |  |
|  | 3.5. During the study period, is the intervention administered (or exposure occurred) as intended? | Yes |  |  |  |
| 1. Quantitative descriptive | 4.1. Is the sampling strategy relevant to address the research question? |  |  |  |  |
|  | 4.2. Is the sample representative of the target population? |  |  |  |  |
|  | 4.3. Are the measurements appropriate? |  |  |  |  |
|  | 4.4. Is the risk of nonresponse bias low? |  |  |  |  |
|  | 4.5. Is the statistical analysis appropriate to answer the research question? |  |  |  |  |
| 1. Mixed methods | 5.1. Is there an adequate rationale for using a mixed methods design to address the research question? | Yes |  |  |  |
|  | 5.2. Are the different components of the study effectively integrated to answer the research question? | Yes |  |  |  |
|  | 5.3. Are the outputs of the integration of qualitative and quantitative components adequately interpreted? | Yes |  |  |  |
|  | 5.4. Are divergences and inconsistencies between quantitative and qualitative results adequately addressed? | Yes |  |  |  |
|  | 5.5. Do the different components of the study adhere to the quality criteria of each tradition of the methods involved? | No |  |  | - As per user guidance, the quality of the qualitative (1.1-1.5) and quantitative components (3.1-3.5) were individually appraised - The qualitative component was rated as high quality (see above) - The quantitative component was rated as moderate quality (see above) - As per user guidance, the overall quality of a mixed methods study cannot exceed the quality of its weakest component – thus overall rating is moderate quality |

Dougherty et al. overall rating: Moderate quality

References (as in manuscript)

55. Hong QN, Pluye P, Fàbregues S, Bartlett G, Boardman F, Cargo M, et al. Mixed methods appraisal tool (MMAT), version 2018 user guide. McGill University Department of Family Medicine. 2018.http://mixedmethodsappraisaltoolpublic.pbworks.com/w/file/fetch/127916259/MMAT_2018_criteria-manual_2018-08-01_ENG.pdf

57. Rasmussen TD, Andersen AN, Ekstrom CT, Jervelund SS, Villadsen SF. Improving health literacy responsiveness to reduce ethnic and social disparity in stillbirth and infant health: A cluster randomized controlled effectiveness trial of the MAMAACT intervention. International Journal of Nursing Studies. 2023;144:104505.

58. Bartlett R, Boyle JA. Developing multi-language maternal health education videos for refugee and migrant women in southeast Melbourne. Midwifery. 2022;111:103369.

59. Dougherty L, Riley A, Caffrey P, Wallbank A, Milne M, Harris MF, et al. Supporting Newly Arrived Migrant Mothers: A Pilot Health Literacy Intervention. Health Lit Res Pract. 2021;5(3):e201-e207.
