# Supplementary material for: Health literacy interventions for pregnant women with limited language proficiency in the country they live in: a systematic review
Source: BMC Public Health. 2024 Nov 26;24:3287. doi: 10.1186/s12889-024-20747-8 (PMC11600627; doi:10.1186/s12889-024-20747-8)
Supplement: Supplementary file 2 — Supplementary Material 2: Summary table of results-table containing a breakdown of each included study. [file 12889_2024_20747_MOESM2_ESM.docx]

Supplementary Table 2, Additional file 2. Summary table of results

| **Author, Year** | **Country** | **Aim** | **Design** | **Target population** | **Study sample** | **Theory/development** | **HL domain** | **Intervention description** | **Outcome measures** | **Key findings** | **Quality of evidence** |
| --- | --- | --- | --- | --- | --- | --- | --- | --- | --- | --- | --- |
| Rasmussen et al. [57] 2023 | Denmark | To reduce ethnic and social disparities in stillbirth and infant death by improving communication between pregnant women and midwives regarding warning signs of pregnancy complications | Cluster randomised controlled trial | Pregnant women in Denmark who were :  18-22 weeks at recruitment  Able to fulfil telephone interviews in Danish, English, Arabic, Turkish, Somali or Urdu  Expected to follow midwife antenatal care visits and to give birth at the hospital of recruitment | 4150 pregnant women, 670 with a Non-Western immigrant background | Based on theories of cultural competence and cultural health capital to guide interpersonal interaction between midwives and pregnant women and health system navigation.  A logic model was developed to illustrate the intervention activities, the expected outcomes, and future impact.  The hypothesis of MAMAACT was that training midwives in cultural competence and intercultural communication and introducing health education materials about when to react to body symptoms and where to go in the healthcare system among pregnant women, would improve communication between pregnant women and midwives regarding pregnancy complications, and thus improve care and perinatal health in the disadvantaged groups. | Active engagement with health care providers  Navigating the health care system  How to respond to pregnancy complications | 6-hour training session for midwives in intercultural communication and cultural competencies  Two dialogue meetings with midwives following training sessions  MAMAACT leaflet and smartphone application in 6 languages on the warning signs of pregnancy complication – distributed to maternity wards | MAMAACT survey – included questions taken from a validated Health literacy questionnaire | No difference was observed in women’s level of active engagement or navigating the healthcare system  Women from the intervention group were more certain of how to respond to complication signs | Moderate |
| Bartlett et al. [58] 2022 | Australia | This research sought to answer the question “how can we use design thinking to create accessible, online resources that are acceptable to women, improve their maternal health awareness, and can be shared effectively through their communities?”  This paper specifically aimed to describe the design process involved with developing animated videos focused on preconception, pregnancy, and postnatal care with women from Arabic- and Dari-speaking communities living in Southeast Melbourne. | Qualitative research. Design thinking methodology | Women who spoke Arabic or Dari as their first language | There was no sample as this study focused on the development of the health literacy intervention | Community user testing sessions with Arabic and Dari speaking women (had used maternity services in Australia previously)  (1) User journey maps and draft posters developed  (2) Posters user tested, scripts developed  (3) Videos developed and user tested  Stage (4) and (5) are implementation/ dissemination/ evaluation stages which were still underway at the time of publishing this article | Knowledge of health and health care systems in pregnancy | Mode of intervention in Arabic and Dari :  (1) Posters  (2) Videos  Content included:  Knowledge of preconception care, early pregnancy support, antenatal care, labour and birth and postnatal care | N/A | Development of 5 posters and 4 videos  Four important recommendations emerged:  (1) Explaining maternity care (appointments, tests, diet, medication etc.)  (2) Culturally informed models of care  (3) Recognising the role of the support person  (4) The impact of Covid-19 on health provision and access  Intervention effectiveness has not yet been assessed in a study sample of pregnant women | Moderate |
| Dougherty et al. [59] 2021 | Australia | To develop and pilot a health literacy intervention, in the form of culturally redesigned new parent classes, in a culturally diverse location in Australia. | Pilot study, mixed methods design | Bangla or Mandarin speaking women who self-identified as a migrant with a baby age 0 to 1 year and who lived in Sydney Local Health District. Grandmothers were also invited to attend the classes | 30 women | New parent classes were developed based on the local health service’s existing new parent class outlines, with some cultural adaptation, prioritisation of select content that occurred through a co-design workshop with Child and family health staff and BCRs as the community representatives | Domain 2 – Having sufficient information to manage health  Domain 4 – Social support for health  Domain 8 – ability to find good health information | A 2-hour session per week over four-week period in Bangla and Mandarin (delivered with an interpreter)  Topics  Adjusting to motherhood  Childhood illnesses  Transitions during parenthood  Where to go for information and in what circumstances | A mixed-methods evaluation was conducted to measure  (1) Recruitment and attendance of participants, (2) Feasibility of the intervention (3) Health literacy of participants,  (4) Provider understanding of barriers to health care access.  Semi-structured interviews post intervention, focus groups post intervention and health literacy questionnaires pre and post intervention | Both Bangla and Mandarin speaking groups had higher health literacy across all three health literacy domains at the post intervention survey compared to pre intervention survey | Moderate |

 References (as in manuscript)

57. Rasmussen TD, Andersen AN, Ekstrom CT, Jervelund SS, Villadsen SF. Improving health literacy responsiveness to reduce ethnic and social disparity in stillbirth and infant health: A cluster randomized controlled effectiveness trial of the MAMAACT intervention. International Journal of Nursing Studies. 2023;144:104505.

58. Bartlett R, Boyle JA. Developing multi-language maternal health education videos for refugee and migrant women in southeast Melbourne. Midwifery. 2022;111:103369.

59. Dougherty L, Riley A, Caffrey P, Wallbank A, Milne M, Harris MF, et al. Supporting Newly Arrived Migrant Mothers: A Pilot Health Literacy Intervention. Health Lit Res Pract. 2021;5(3):e201-e207.
